# Evaluating the effects of glutaraldehyde concentration and incubation time on the structural integrity of the human pericardium

**DOI:** 10.3389/fbioe.2026.1746518

**Published:** 2026-06-17

**Authors:** Sahra Tasdelen, Gianluca Dimonte, Irna Cutuk, Andreas Teuschl-Woller, Thomas Poschner, Severin Laengle, Viktoriia Tymoshenko, Barbara Messner, Thomas Augustin, Lena Hirtler, Martin Andreas

**Affiliations:** 1 Christian Doppler Laboratory for Microinvasive Heart Surgery, Department of Cardiac Surgery, Medical University of Graz, Graz, Austria; 2 Christian Doppler Laboratory for Microinvasive Heart Surgery, Department of Cardiac – and Thoracic Aorta Surgery, Medical University of Vienna, Vienna, Austria; 3 Department Life Science Engineering, University of Applied Sciences Technikum Wien, Vienna, Austria; 4 Cardiac Surgery Research Laboratory, Department of Cardiac – and Thoracic Aorta Surgery, Medical University of Vienna, Vienna, Austria; 5 Institute for Medical Informatics, Statistics and Documentation, Medical University of Graz, Graz, Austria; 6 Division of Anatomy, Centre for Anatomy and Cell Biology, Medical University of Vienna, Vienna, Austria

**Keywords:** biomaterials, cardiac surgery, mechanical properties, pericardium, tissue processing

## Abstract

**Background:**

Glutaraldehyde (GA) fixation of pericardial tissue is widely used in bioprosthetic heart valves. However, how GA concentration and incubation time jointly influence structure-function relationships remains incompletely understood. This study systematically evaluates the effects of GA concentration and incubation time on collagen architecture and mechanical behavior in human pericardial tissue.

**Results:**

Human pericardium was treated with GA concentrations of 0%–2.5% for 5–90 min. Collagen structure was quantified using Picrosirius red imaging and image analysis, while mechanical behavior was assessed via strain-controlled uniaxial tensile testing. Increasing incubation time was associated with reduced collagen waviness and evidence of fiber bundling. Mechanical analysis revealed a significant interaction between GA concentration and incubation time for maximum strain and transition strain (p < 0.05), indicating a non-linear, non-additive treatment effect. Repeated-measures correlation analysis showed strong internal consistency among mechanical parameters (e.g., maximum stress vs. high-strain modulus, r_rm_ = 0.92), whereas structural descriptors such as waviness and fiber orientation exhibited minimal association with mechanical outcomes (r_rm_ = 0.06).

**Conclusion:**

GA treatment effects arise from a coupled, non-additive interaction between concentration and incubation time rather than a simple dose-response relationship. Importantly, commonly used structural metrics do not directly predict mechanical performance, underscoring the need for integrated structure-function assessment. These findings provide a framework for future evaluation of GA-treated pericardium in bioprosthetic applications.

## 1 Background

Since the 1960s, GA has played a central role in biological tissue preservation and surgical practice (Carpentier et al., 1969). Originally developed and introduced as a disinfectant and sterilizing agent, GA has been widely used to disinfect surgical equipment, endoscopes, and other reusable medical instruments due to its broad-spectrum antimicrobial activity and ability to inactivate spores, bacteria and viruses (Johnson et al., 1982; Cronmiller et al., 1999). Its utility extended beyond disinfection to the biopreservation of biological tissues, driven by its ability to chemically stabilize proteins and structural matrices by the formation of covalent bonds between amino groups (Jayakrishnan and Jameela, 1996)

On a molecular level, GA ((CH_2_)_3_(CHO)_2)_, is an amine-reactive homobifunctional reagent, that forms covalent crosslinks between free amino groups, especially those on lysine (Lys) residues in collagen and elastin, leading to tissue stabilization. The two carbonyl ends of GA form Schiff’sche bases with the positively charged amino groups on the surface of the proteins. Enzymatic crosslinking of proteins begins with the oxidation of Lys or hydroxylysine (Hyl) residues by lysyl oxidase, generating reactive aldehyde groups. These aldehydes then undergo spontaneous condensation reactions with other Lys, Hyl, or aldehyde groups to form immature covalent crosslinks, which subsequently mature into stable, multivalent crosslinks that contribute to the structural integrity of, e.g., the extracellular matrix. Monomeric glutaraldehyde polymerizes by condensation, giving rise to mixtures of elongated species that can in turn also crosslink intramolecular- and/or intermolecular lysines in a nonspecific manner (Migneault et al., 2004; Gaar et al., 2020; Salem et al., 2010).

In collagen, not all amine groups are available for the GA reaction, with a ratio of five amino acid residues per 1000 total residues within 24 hrs. When combined with a too high concentration and too long crosslinking reaction time, it can decrease the crosslinking density and add free aldehydes to the matrix. Further, GA is capable of self-polymerizing and forming three-dimensional networks, which may further alter the structural properties of the crosslinked matrix (Rýglová et al., 2017). A consequence could be the trigger of inflammatory responses and promoting calcification by providing nucleation sites. Collagen molecules (molecular weight ∼300,000 Da) are organized into highly ordered fibrillar structures, consisting of rod-like triple helices, approximately 300 nm in length and 1.5 nm in diameter. These molecules exhibit a high degree of axial alignment and a characteristic staggered arrangement, with each collagen molecule offset by approximately one-quarter of its length relative to its neighbor. Alterations at the molecular level, such as changes in crosslinking chemistry or molecular conformation, can disrupt this hierarchical organization, thereby compromising fibrillar architecture and reducing the overall mechanical integrity of the tissue (Nimni and Harkness, 2018). This introduces undesirable side effects, such as increased tissue stiffness, reduced biocompatibility, and calcification (Vyavahare et al., 1999). These limitations have prompted ongoing investigations into optimization strategies, including reduced concentrations, altered incubation times, or combination treatments, to balance the durability and physiological compliance. Although glutaraldehyde is known to crosslink both collagen and elastin, this study focuses on collagen due to its predominance in pericardial tissue and its primary role in determining mechanical properties, as well as its higher reactivity with glutaraldehyde compared to elastin (Cohn et al., 1987).

The innovative introduction of GA in cardiovascular surgery came in the late 1960s and early 1970s through Alain Carpentier and colleagues, who used it to crosslink collagen fibers in porcine and bovine tissues and thereby preserving and strengthening biological heart valves for implantation (Carpentier et al., 1969; Carpentier et al., 1982). GA fixation became a gold standard in the manufacture of bioprosthetic heart valves, reducing antigenicity, halting enzymatic degradation, and improving mechanical durability (Lim et al., 2012; Schoen and Levy, 1997).

GA-treated tissues have also been used in other surgical domains beyond cardiovascular reconstruction, such as vascular grafts, pericardial patches for congenital heart repair, and reconstructive surgery for reinforcing soft tissues, in both adult and pediatric patients (Neethling et al., 2014; Inoue et al., 2021; Seguin et al., 2009; Gluck et al., 2022). These diverse applications underline the need for a deeper understanding of how GA treatment parameters affect the mechanical and biological behavior of tissues.

In recent years, autologous bioprosthetic approaches gained more interest, particularly techniques such as the Ozaki procedure, where patient-derived pericardium is treated with GA (typically at 0.6% for around 10 min) before being fashioned into functional aortic valve cusps (Ozaki et al., 2014). Despite the clinical success of such procedures, the rationale behind the chosen GA concentration and exposure time seems to be often empirical (Maurer et al., 2018; Korossis et al., 2002) and not well-characterized, especially in the context of human pericardial tissue, which differs structurally from commonly studied animal models (Cohn et al., 1987; Maurer et al., 2018).

This study aims to address that gap by systematically exploring how different GA concentrations and incubation durations affect the structural and mechanical properties of human pericardium, using both image-based analysis and tensile testing. Although numerous studies have examined GA-treated animal tissues, relatively few have focused on human-derived materials, which are increasingly relevant for patient-specific valve reconstruction and personalized cardiovascular surgeries.

Our approach revisits the unresolved questions posed by Carpentier et al. in 1974: “*What is the host reaction to differently treated tissue? Is the effect of host cell ingrowth into the valve beneficial or harmful? What is the long-term fate of collagen and elastin? And, finally, what is the adequate method for preservation of such grafts*?”

While this study does not aim to address immunogenicity or calcification, it provides experimental insight into the mechanical consequences of modulating GA treatment in human pericardium. It is an exploratory investigation, intended to generate hypotheses and provide a reproducible framework for further refinement of tissue engineering strategies involving crosslinking agents, such as GA. We hypothesized that, despite numerous studies, the effects of commonly used GA fixation concentrations in human tissues remain insufficiently characterized due to the complexity of the system, and that varying both concentration and incubation time may reveal important mechanical differences.

## 2 Methods

Pericardial tissue was obtained from six different human donors, including three surgical patients and three recently deceased donors. Multiple tissue strips were prepared per donor and subjected to different treatment conditions. All samples were processed and analyzed under standardized protocols to minimize variability and ensure comparability across groups.

### 2.1 Sample preparation

Human pericardial tissue was obtained from six donors, including three patients undergoing cardiac surgery (intraoperative samples) and three recently deceased donors. Following collection, samples were processed as soon as feasible. In cases where immediate processing was not possible, tissue was temporarily stored in Dulbecco’s Modified Eagle Medium (DMEM) under controlled conditions (37 °C, 5% CO_2_) and processed within 48 h.

Pericardial tissue was cut into rectangular strips measuring approximately 5 mm × 20 mm using a scalpel and ruler to ensure consistent dimensions. Due to variability in the size and geometry of the harvested tissue, strips were prepared to maximize usable material while maintaining comparable dimensions. Where possible, the long axis of each strip was aligned with the predominant visible collagen fiber orientation. Particular attention was given to the preparation of samples designated for uniaxial tensile testing: these were trimmed to have consistent shape and size, with dimensions (width and length) measured using a ruler to ensure comparability across conditions. The remaining half of the tissue, for which exact dimensional consistency was less critical, was allocated for histological analysis. Histological analyses were performed on samples from all donors. Uniaxial tensile testing was conducted exclusively on intraoperative samples (n = 3 donors) to minimize the influence of post-mortem changes on mechanical properties.

Following GA treatment, samples designated for histological analysis were fixed in 4.5% formalin and embedded in paraffin for subsequent sectioning and staining (see Section 2.3). Samples intended for mechanical testing were immersed in phosphate-buffered saline (PBS; without calcium and magnesium, Gibco) and stored at 4 °C until testing. Although storage duration varied due to logistical constraints, all samples were maintained under identical conditions and tested within a defined time window after treatment.

### 2.2 Glutaraldehyde treatment

Each tissue was treated with GA at concentrations ranging from 0.1% to 1.0% in 0.1% increments, and from 1.0% to 2.5% in 0.5% increments and one stripe was left out for control. The incubation times ranged from 5, 10, 20, 30, 60 and 90 min. GA dilutions were prepared from a 50% GA stock solution (Sigma Aldrich, Cat. No. 340855) in 1x PBS ([-] Calcium Chloride [-] Magnesium Chloride) at room temperature and thoroughly mixed using a vortex mixer. GA dilutions were freshly prepared before each use. Individual samples were incubated in separate wells of a 6-well cell culture plate, each containing approximately 5 mL of the respective GA solution.

### 2.3 Histopathological analysis and staining

After fixation in 4.5% formalin, samples were embedded in paraffin and sectioned at 3 µm thickness using a microtome (Leica). Sections were stained with Picrosirius red (Merck, Cat. No.: 365,548) following the manufacturer’s instructions. Imaging was performed using either a point scanning microscope (Nikon Eclipse Ti Series), or a slide scanner (Pannoramic 250 Flash III, 3DHISTECH). Image analysis was conducted using CaseViewer (3DHISTECH) and ImageJ (NIH). From the stained sections, collagen fiber architecture and orientation were evaluated.

### 2.4 Image acquisition and analysis

**FIGURE 1:**
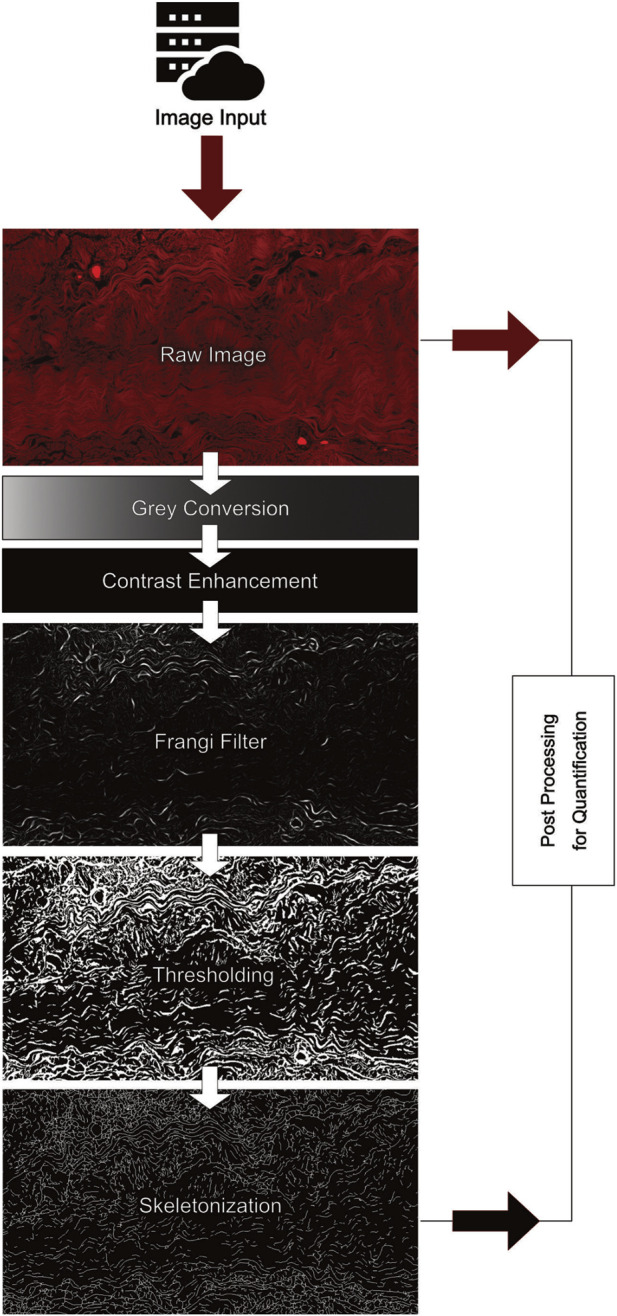
Schematic representation of the image analysis pipeline. The process begins with the raw image input, which undergoes grayscale conversion followed by contrast enhancement. A fringe filter is then applied, after which thresholding and skeletonization are performed. Both the raw image and the skeletonized data are subsequently used for post-processing and quantitative analysis.

Each image was analyzed using the Python library scikit-image (skimage), utilizing the skeletonize function as part of the processing pipeline. The image analysis workflow comprised the following steps, which are described below and visually summarized in Figure 1.

Conversion of the input image to a binary format:

1. Contrast Enhancement - Adjustment of image contrast to improve feature visibility
2. Frangi Filter Application - Enhancement of fiber-like structures using the Frangi vesselness filter
3. Thresholding and Removal of Small Objects - Pixel intensities were thresholded at the 70th percentile, and objects smaller than 10 pixels were removed to reduce noise
4. Skeletonization - Reduction of fiber structures to one-pixel-wide centerlines using the skeletonize function
5. Quantification - Measurement of fiber length and orientation from the resulting skeletons

**FIGURE 2:**
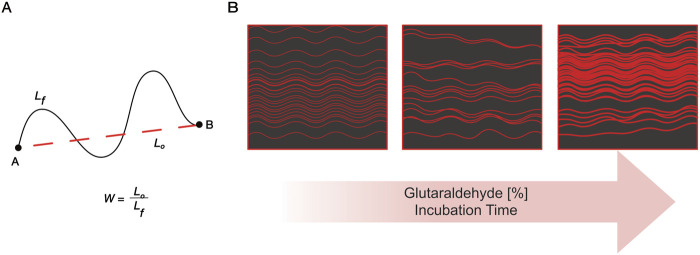
Quantification of collagen waviness and schematic representation of treatment effects. **(A)** Measurement of Euclidean distance in a wavy structure. The straight-line distance between endpoints A and B represents the Euclidean distance [L_0_], while the actual path length [L_f_] follows the undulating contour of the structure. The difference between these lengths reflects the degree of waviness [W], with increased undulation resulting in a longer path length relative to the straight-line distance. **(B)** Schematic illustration of the hypothesized effect of glutaraldehyde (GA) treatment on collagen architecture. From left to right, increasing GA concentration and incubation time (indicated by the arrow) are associated with progressive collagen fiber bundling and reduced waviness. Collagen fibers are depicted in red, transitioning from loosely organized, wavy structures to more aligned, compact bundles, representing a reduction in crimp and increased structural organization.

For each identified fiber, orientation angle and length were calculated. Orientation is defined as the angle between the 0th axis (rows) and the major axis of the ellipse with the same second moments as the fiber region, ranging from - π/2 to π/2 counterclockwise. The Euclidean distance between the starting and the ending point of each fiber is also calculated to determine the Waviness index (W). W is calculated as the ratio between minimum distance and the actual distance of the fiber, as seen in Figure 2A and calculated using Equation 1.

$$W=\frac{Lo}{Lf}$$ (1)

W ranges from 0 to 1, where a value of one means “straight fiber”. Each image identified multiple fibers and W is calculated for each image. The mean value of W, along with the fiber orientation, is then used as a feature for further analysis (Niestrawska et al., 2022).

To evaluate collagen fiber straightening and fiber bundling (Figure 2B), red and black pixel of the image were evaluated. The red-to-black pixel ratio was used as an exploratory image-derived metric to describe threshold-based changes in staining appearance under standardized RGB settings. This metric was not intended as a validated direct measure of collagen bundling, but as a supplementary descriptive parameter to support qualitative image assessment.

Pixel classification was used as a surrogate measure for collagen bundle density in Picrosirius red-stained sections. Red and black pixels are counted according to the following RGB masks:

Red → R > 100 G < 80 B < 80

Black → R < 30 G < 30 B < 30

The fractions of red and black pixels are calculated, along with the ratio between red and black pixel fractions (RB ratio). An R/B ratio close to one indicates that red and black pixels are present in roughly equal amounts. A low ratio suggests that black pixels predominate, which corresponds to a higher degree of fiber bundling. A high R/B ratio indicates that fibers are minimally crosslinked. It is important to note that the red and black pixel fractions are not complementary, as some pixels fall outside both red and black masks due to variations in intensity gradients within each channel.

### 2.5 Uniaxial tensile testing

Prior to uniaxial tensile testing (Zwick Roell Z005, Zwick GmbH and Co., KG, Ulm, Germany), samples were stored immersed in 1x PBS ([-] Calcium Chloride, [-] Magnesium Chloride) at 4 °C. The rectangular specimens were gently blotted to remove excess surface liquid to facilitate uniform gripping and to avoid the sample from slippage, then mounted between the clamps of the tensile tester (Supplementary Figure S1). Tissue specimens were preloaded to 0.01 N and subjected to 7 preconditioning loading-unloading cycles at 15% maximum strain, with an elongation rate of 10 mm/min. Curves fifth to seventh of these preconditioning cycles became nearly superimposable indicating a mechanically stable step by minimizing viscoelastic effects and overall reducing sample variability. Following preconditioning, specimens were again preloaded to 0.01 N, the initial length (L_0_) was measured, and uniaxial tensile testing was performed to failure at a constant rate of 10 mm/min. The samples were kept fully hydrated with 1x PBS ([-] Calcium Chloride, [-] Magnesium Chloride) throughout the entire test, which was conducted at room temperature (Jonas et al., 2002). The thickness of each specimen was determined from microscopic images by measuring five distinct regions along the same tissue strip (See Additional File 6). The mean value of these five measurements was used to calculate the mechanical parameters.

The testing machine acquired the tensile force F and directly the strain ε , according to the definition of Equation 2:

$$\varepsilon =\frac{\left(l-l_{0}\right)}{l_{0}}$$ (2)

Here, $l_{0}$ is the initial length of the specimen measured between the clamps, prior to the application of strain. Subsequently, the engineering stress ( σ ) is calculated according to the definition of Equation 3:

$$\sigma =\frac{F}{A}$$ (3)

The cross-sectional area *A* was calculated as in Equation 4:

A=w ·t (4)

where w is the width and t is the thickness.

A stress-strain curve is then calculated and described by the means of six key parameters used to evaluate the behavior of biological soft tissue: maximum tensile stress ( $\sigma_{\max}$ ) and strain ( $\varepsilon_{\max}$ ), elastic modulus at low strain ( $E_{low}$ ), elastic modulus at high strain ( $E_{high}$ ), transition stress ( $\sigma_{trans}$ ) and strain ( $\varepsilon_{trans}$ ). A representative graph of the stress-strain curve is shown in Figure 5A.

$\sigma_{\max}$ corresponds to the maximum stress and $\varepsilon_{\max}$ is the respective strain at that point. Linear regression is then performed in two separated strain ranges; one at low strain [2%; $\left.\varepsilon_{trans}\right]$ , where elastin behavior is dominant, and one at high strain [ $\left.\varepsilon_{trans\;};\;\varepsilon_{\max}\;\right]$ , where the collagen behavior is predominant. In the transition phase gradually more collagen fibers align and therefore increase the elastic modulus. The transition point is found by minimizing the combination of residual sum of squares (RSS) of the regression in the two-strain range (Equation 5):

$$RSS=\sum_{i=1}^{n}{\left(y_{i}-{\hat{y}}_{i}\right)}^{2}$$ (5)

Where $y_{i}$ is the observed value and ${\hat{y}}_{i}$ is the predicted value from the regression model. The slope of each linear regression corresponds to the elastic modulus in megapascals (MPa) (Korossis et al., 2002; Vinci et al., 2013).

### 2.6 Statistical analysis

Data were analyzed using the two-way analysis of variance (ANOVA) to evaluate the effects of treatment concentration, incubation time, and their interaction. Prior to performing ANOVA, the assumptions of normality and homogeneity of variances were assessed and found to be met. When significant differences were detected, *post hoc* pairwise comparisons were conducted using Tukey’s and/or Bonferroni corrections to account for multiple comparisons. Statistical significance was set at *p* < 0.05. All data analysis was performed using Python (version 3.11.9) along with the following libraries: NumPy (2.0.0), pandas (2.2.2), matplotlib (3.9.0), SciPy (1.14.1), scikit-learn (1.6.1), statsmodels (0.14.4), and scikit-image (0.25.2). For detailed functionality and usage, please refer to the respective official documentation.

To account for the non-independence of repeated measures (15 observations per subject), we calculated the repeated measures correlation (r_rm_) using the rmcorr-package in R. This method accounts for inter-individual differences by removing the between-patient variance and estimating the common intra-individual association. Additionally, the pairwise relationships between mechanical parameters were analyzed by means of linear mixed-effects (LME) models. To account for between-subject variance, a random intercept for subject was included. The model was estimated using Restricted Maximum Likelihood (REML). The level of significance was set at 0.05 for all tests. To highlight the most robust associations, only regressions with a repeated measures correlation of |r_rm_| ≥ 0.70 were included as plots. Data were analyzed using R (Version 4.5.2) in the RStudio environment (Version 2026.01.0).

## 3 Results

### 3.1 Tissue preparation and visual assessment of fiber thickness

**FIGURE 3:**
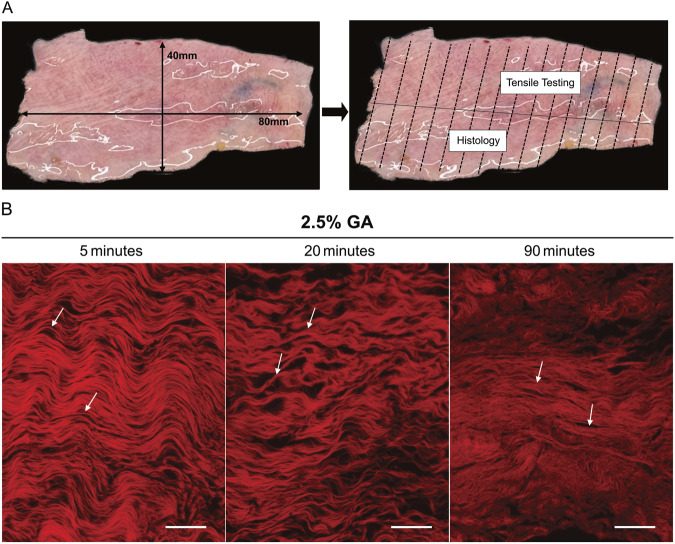
Pericardial processing with glutaraldehyde (GA) at different incubation times. **(A)** Representative image of human pericardium prior to treatment. Black arrows indicate the initial width (80 mm) and height (40 mm). The dotted lines indicate the cutting direction used for sample preparation, based on specimen geometry rather than predefined collagen fiber orientation. Half of the sample was allocated for histological analysis and the other half for tensile testing. Untreated tissue served as the baseline control (0% GA, 0 min incubation) for all subsequent analyses. **(B)** Pericardial tissue sections treated with 2.5% GA at different incubation times (5, 20, and 90 min, from left to right), stained with Picrosirius red and imaged using confocal microscopy. Images represent different regions of the same pericardial specimen for each condition, rather than the same region imaged over time. White arrows indicate collagen fiber alignment. Scale bar: 100 μm.

Figure 3A shows a representative image of the harvested pericardium after excision, including its approximate dimensions and preparation steps prior to treatment (40 mm × 80 mm). Figure 3B displays representative treated pericardial samples from the group treated with 2.5% GA at different incubation times. The same tissue region was imaged at different time points to allow direct assessment of temporal structural changes under GA exposure. Collagen fiber alignment, marked by arrows, was visually assessed. Although no quantitative image analysis was conducted on these specific features, qualitative differences in collagen fiber orientation and morphology were observed across incubation conditions. In samples fixed for 5 min (Figure 3B, left panel), the fibers appeared thin and densely packed. With increasing incubation time, the fibers became progressively thicker (Figure 3B, middle and right panel). Concurrently, a qualitative reduction in collagen waviness was observed, consistent with the hypothesis that increasing GA concentration and exposure duration promote fiber bundling and straightening. Notably, it was reassuring to observe that the collagen fibers retained their axial alignment and characteristic staggered arrangement across all conditions. These observations, while subjective, suggest treatment-related alterations in tissue microstructure and support further quantitative evaluation in future work.

### 3.2 ECM structural analysis and correlation analysis

**FIGURE 4:**
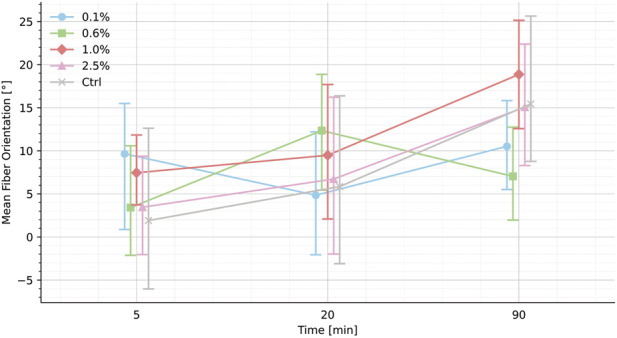
Fiber Orientation Analysis of glutaraldehyde (GA) treated human pericardium. **(A)** Interaction plot of fiber orientation [degree°] in dependence of different Glutaraldehyde (GA) concentrations (Control (Ctrl)…grey and marked with x; 0.1%…blue and marked with ●; 0.6%…green and marked with ■; 1.0%.red marked with ◆ and 2.5%…pink marked with ▲) and incubation times (5, 20 and 90 min). A consistent increase in the mean orientation across all concentrations, with no systematic differences between the concentration groups is shown. Each data point represents the mean of six biological replicates (patient samples), the error bars show the standard deviation.

An interaction plot (Figure 4) was used to examine the combined effects of incubation time and treatment concentration on pericardial fiber orientation, expressed in degrees. The x-axis displays the three incubation times (5, 20, and 90 min), while the y-axis shows the mean fiber orientation angle. Each line represents the mean of different GA concentrations (Control, 0.1%, 0.6%, 1.0% and 2.5%). The crossing of lines across time points suggests a potential interaction effect, where the impact of concentration on fiber alignment may vary depending on incubation duration. Table 1 presents the corresponding two-way ANOVA results.

**TABLE 1:** Two-Way anova of fiber orientation. Table summarizing results from the two-way ANOVA, showing the effects of incubation time, treatment concentration, and their interaction (time × concentration). Reported values include degrees of freedom (df), F-values, and p-values.

Effect	df	F-Value	p-Value
Incubation time [min]	2.0	4.84	0.01*
GA [%]	4.0	0.62	0.65
Interaction (time × GA)	8.0	0.99	0.46
Residual	69	–	–

Bold values and asterisks are used to highlight statistically significant values, defined as p < 0.05.

The orientation describes the average angle of the fibers relative to the horizontal and serves as a measure of the structural organization of the extracellular matrix. The analysis showed a significant effect of the incubation time on the fiber orientation (p < 0.05). With increasing incubation time, orientation values increased in all concentration groups, indicating a time-dependent change in fiber orientation. The highest mean values were observed after 90 min of incubation, while the lowest orientations were observed after 5 min. While this indicates that fiber orientation is influenced by treatment duration, *post hoc* comparisons did not reveal statistically significant pairwise differences between individual time points, likely due to variability and limited sample size (n = 3). These results suggest fiber reorganization over time, but further analysis with larger cohorts would be needed to confirm specific time-dependent effects. Neither the GA concentration alone (p > 0.05) nor the interaction between concentration and time (p > 0.05) had a significant effect on orientation. These findings suggest that temporal effects dominate over concentration-dependent effects, although interpretation is limited by the inherent heterogeneity of human pericardial tissue.

### 3.3 Uniaxial tensile testing

Different mechanical parameters derived from uniaxial tensile testing, including maximum stress, maximum strain, low-strain elastic modulus (E_low_), high-strain elastic modulus (E_high_), transition strain, and transition stress, were evaluated across the GA concentrations of 0.1%, 0.6%, 1.0% and 2.5% and incubation times from 5 min, 20 min up to 90 min. Untreated samples (0% GA) were used as the baseline control condition, corresponding to the 0 min exposure state. For each concentration, data from three incubation durations are shown. All treatment groups were compared to the untreated control (1x PBS without Ca^++^ and Mg^++^), with statistical variation indicated by error bars.

#### 3.3.1 Maximum strain

**FIGURE 5:**
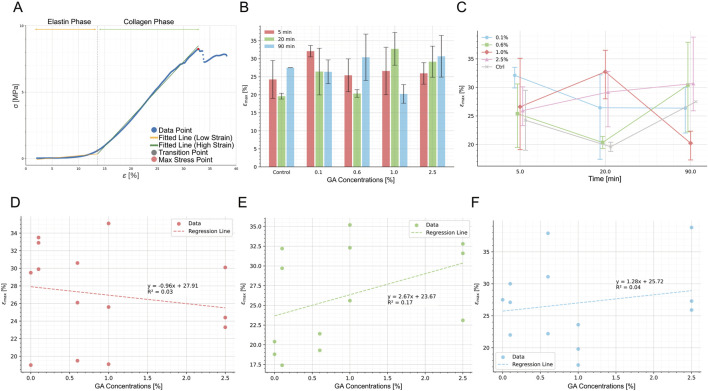
Effect of treatment time and concentration on maximum strain in tensile testing. **(A)** Representative stress–strain curve from uniaxial tensile testing. Stress (σ) is expressed in MPa and strain (ε) in %. Blue dots indicate measured data points. The elastin-dominated phase is highlighted in orange with a dotted line representing the linear fit in the low-strain region, while the collagen-dominated phase is highlighted in green with a dotted line representing the linear fit in the high-strain region. The grey dot marks the transition point (εtrans) between the two phases, and the red dot indicates the maximum stress point (σmax). **(B)** Barplot showing the maximum strain (Emax) across different Glutaraldehyde (GA) concentrations. Each bar represents the mean from all patients treated for 5 min (red), 20 min (green), or 90 min (blue) at the respective GA concentrations. Error bars indicate variability (standard deviation) across patients (n = 3). **(C)** Interaction plot showing the effects of treatment time and concentration (Control (Ctrl)…grey and marked with x; 0.1%…blue and marked with ●; 0.6%…green and marked with ■; 1.0%.red marked with ◆ and 2.5%…pink marked with ▲) on maximum strain. Each data point represents the mean of three biological replicates (patient samples), the error bars show the standard deviation. **(D–F)** Linear regression plots showing the relationship between GA concentration and maximum strain (Emax) for each incubation time: **(D)** 5 min (red), **(E)** 20 min (green), **(F)** 90 min (blue). In each plot, the x-axis represents increasing GA concentrations, and the y-axis shows the corresponding maximum strain. Trend lines illustrate the linear relationship for each time point.

All mechanical parameters were derived from the initial uniaxial tensile test conducted to failure. A representative stress–strain curve is presented in Figure 5A to facilitate visualization of the data. To evaluate the effect of GA concentration and incubation time on the pericardial mechanical response, maximum strain was analyzed across conditions. Bar plots comparing different GA concentrations (Figure 5B) reveal that strain values vary substantially across groups, with no apparent uniformity across incubation times within a given concentration. This suggests that both concentration and time contribute to the mechanical behavior of the tissue. To further investigate whether these factors interact, an interaction plot was generated (Figure 5C), with incubation time on the x-axis and maximum strain on the y-axis. Each line represents pooled values (n = 3) for a given GA concentration. The observed crossover between lines indicates a potential interaction effect, where the influence of concentration depends on the incubation duration. Statistical analysis using the two-way ANOVA (Table 2) confirmed a significant interaction between treatment concentration and incubation time (*p* < 0.05) on the maximum strain.

**TABLE 2:** Two-Way ANOVA: effect of treatment time and concentration on maximum strain. Table summarizing results from the two-way ANOVA, showing the effects of incubation time, treatment concentration, and their interaction (time × concentration) on the maximum strain. Reported values include degrees of freedom (df), F-values, and p-values.

Effect	df	F-Value	p-Value
Incubation time [min]	2.0	0.13	0.8756
GA [%]	3.0	0.35	0.7868
Interaction (time × GA)	6.0	2.51	0.0497*
Residual	24.0	–	–

Bold values and asterisks are used to highlight statistically significant values, defined as p < 0.05.

However, subsequent *post hoc* tests did not identify statistically significant pairwise differences between specific groups, likely due to variability within conditions and limited sample size. This highlights the influence of tissue heterogeneity, which may obscure group-specific effects despite an overall significant interaction.

The regression analysis of the maximum strain further exhibited group-specific trends in response to increasing GA concentrations (Figures 5D–F). In the 5-min incubation group (Figure 5D), a slight downward trend was observed (slope = −0.96, *R* ^2^ = 0.03), indicating minimal reduction in strain with higher concentrations. In contrast, samples incubated for 20 min showed a stronger upward trend (slope = 2.67, *R* ^2^ = 0.17), suggesting a concentration-dependent increase in strain (Figure 5E). For the 90-min group, the trend continued upward but with a less steep slope, indicating a more moderate increase in maximum strain compared to the 20-min group (Figure 5F). While none of the models demonstrated high explanatory power, these trends may point to time-dependent differences in tissue mechanical response (Table 3).

**TABLE 3:** Regression parameters for maximum strain by incubation time: slope, intercept, and R^2^. Summary table of regression parameters, including slope, intercept, and coefficient of determination (R^2^) for each incubation time. Slope is expressed as % strain per % concentration. R^2^ is unitless.

Time point	Slope	Intercept	*R* ^2^ Score
5 min	−0.96	27.91	0.028
20 min	2.44	24.78	0.112
90 min	1.28	25.72	0.035

#### 3.3.2 Transition strain

**FIGURE 6:**
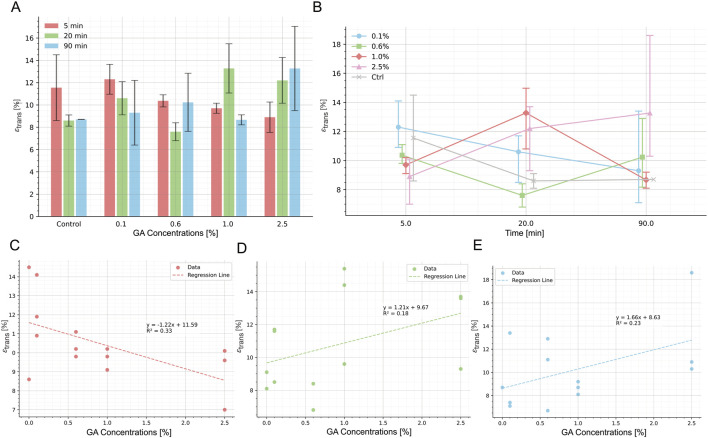
Effect of treatment time and concentration on transition strain in tensile testing. **(A)** Barplot showing the transition strain (εtrans) across different Glutaraldehyde (GA) concentrations. Each bar represents the mean from all patients treated for 5 min (red), 20 min (green), or 90 min (blue) at the respective GA concentrations. Error bars indicate variability (standard deviation) across patients (n = 3). **(B)** Interaction plot showing the effects of treatment time and concentration (Control (Ctrl)…grey and marked with x; 0.1%…blue and marked with ●; 0.6%…green and marked with ■; 1.0%.red marked with ◆ and 2.5%…pink marked with ▲) on transition strain. Each data point represents the mean of three biological replicates (patient samples), the error bars show the standard deviation. **(C–E)** Linear regression plots showing the relationship between GA concentration and transition strain (εtrans) for each incubation time: **(C)** 5 min (red), **(D)** 20 min (green), **(E)** 90 min (blue). In each plot, the x-axis represents increasing GA concentrations, and the y-axis shows the corresponding transient strain. Trend lines illustrate the linear relationship for each time point.

The influence of GA treatment duration and concentration on transition strain was further assessed across all conditions. Bar plots summarizing the transition strain across all treatment conditions are shown in Figure 6A. Each bar represents the mean value for a given GA concentration, with incubation time variations (5, 20, and 90 min) displayed within each group. Error bars indicate variability across samples, and the noticeable differences in strain between groups suggest a time- and dose-dependent response. To explore potential interactions between incubation time and concentration, an interaction plot was generated (Figure 6B). The crossing of lines indicates that the effect of one factor may depend on the level of the other, suggesting an interaction between treatment parameters. This observation was supported by two-way ANOVA (Table 4), which revealed a statistically significant interaction between incubation time and GA concentration (*p* < 0.05).

**TABLE 4:** Two-Way ANOVA: effect of treatment time and concentration on transition strain. Table summarizing results from the two-way ANOVA, showing the effects of incubation time, treatment concentration, and their interaction (time × concentration). Reported values include degrees of freedom (df), F-values, and p-values.

Effect	df	F-Value	p-Value
Incubation time [min]	2.0	0.62	0.55
GA [%]	3.0	0.74	0.54
Interaction (time × GA)	6.0	2.55	0.0472*
Residual	24.0	–	–

Bold values and asterisks are used to highlight statistically significant values, defined as p < 0.05.

However, similar to the findings for maximum strain, *post hoc* pairwise comparisons did not reveal statistically significant differences between specific groups. This reflects variability within conditions or a limited sample size (n = 3). Nevertheless, the significant interaction supports the conclusion that transition strain is jointly influenced by both treatment concentration and duration in a non-additive manner. This again reflects the high variability of biological tissue and the limited sample size (n = 3), which reduce statistical power.

To further explore how treatment concentration influences transition strain across different GA incubation times, a regression analysis was performed. For each incubation time point - 5, 20, and 90 min - a linear regression model was fitted to assess the relationship between GA concentration and transition strain. The resulting trend lines are shown in Figures 6C–E, each representing one of the time points: 5 min (red), 20 min (green), and 90 min (blue). These plots reveal time-dependent differences in how transition strain responds to increasing GA concentration. The 5-min incubation group showed a decreasing trend in transition strain with increasing GA concentration (slope = −1.22, *R* ^2^ = 0.33), which may suggest earlier collagen fiber alignment under mechanical load (Figure 6C). In contrast, the 20-min group demonstrated an upward trend (slope = 1.21, *R* ^2^ = 0.18), indicating delayed alignment with higher concentrations (Figure 6D). Similarly, the 90-min group exhibited a continued increase in transition strain (slope = 1.66, *R* ^2^ = 0.23), as seen in Figure 6E. Although the explanatory power of the models remains modest and low in exploratory power (Table 5), the trends suggest a time-dependent shift in the mechanical response related to fiber recruitment. Detailed subject-specific regression analyses based on linear mixed-effects models are provided in the Supplementary Material to support these findings, as well as additional mechanical parameters that did not reach statistical significance for completeness.

**TABLE 5:** Regression parameters for transition strain by incubation time: slope, intercept, and R^2^. Summary table of regression parameters, including slope, intercept, and coefficient of determination (R^2^) for each incubation time. Slope is expressed as % strain per % concentration. R^2^ is unitless.

Time point	Slope	Intercept	*R* ^2^ Score
5 min	−1.22	11.59	0.332
20 min	1.06	10.04	0.129
90 min	1.66	8.63	0.228

### 3.4 Correlation analysis

**FIGURE 7:**
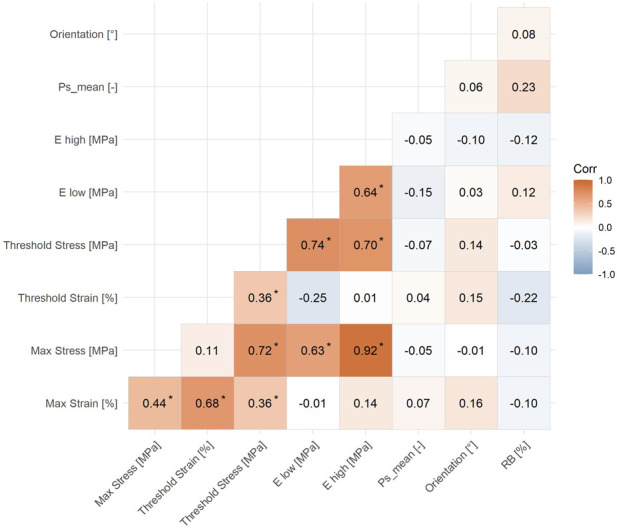
Within-person repeated-measures correlations among structural and mechanical parameters. Heatmap of within-person correlations (rmcorr) between structural and mechanical variables across all treatment conditions. Each cell represents the repeated-measures correlation coefficient (r) between two variables, accounting for non-independence of observations within the same sample. The color scale ranges from −1.0 (blue, strong negative correlation) to +1.0 (red, strong positive correlation). Asterisks (*) indicate statistically significant correlations (p < 0.05).

To further assess relationships between structural and mechanical parameters, a within-person repeated-measures correlation (rmcorr) analysis was performed (Figure 7; Carpentier et al., 1969). The resulting heat map illustrates pairwise associations across all variables. Strong positive correlations were primarily observed among mechanical parameters, including maximum stress and high-strain elastic modulus (E_high_; r_rm_ = 0.92), as well as threshold stress and low-strain elastic modulus (E_low_; r_rm_ = 0,74). In contrast, structural parameters such as collagen waviness and fiber orientation showed weak or negligible correlations with mechanical outcomes (Range r_rm_: −0.22–0.23). Notably, the correlation between waviness and fiber orientation was minimal (r_rm_ = 0.06), indicating that these parameters behave largely independently under the tested conditions.

## 4 Discussion

This study investigated the effects of varying glutaraldehyde concentrations and incubation durations on the mechanical and structural properties of human pericardial tissue. Across all tested conditions, GA treatment led to measurable changes in key structural and mechanical parameters, including fiber orientation, maximum stress, maximum strain, elastic moduli, and collagen waviness, with trends emerging between the different incubation groups.

This topic may appear well-established, dating back to the 1960s, and is often regarded as a settled issue, especially since it serves as a gold standard in bioprosthesis applications and tissue fixation during adult- and pediatric cardiac surgery (Carpentier et al., 1969; Gluck et al., 2022). However, a closer examination of the literature reveals that it is far from fully understood. Standard practice for the preparation of autologous GA-treated pericardium in cardiovascular interventions involves securing the tissue flat during fixation to prevent shrinkage and edge curling, by clipping it to a stable surface. Fixation times commonly range from 15 to 30 min, depending on the intended surgical application with a GA concentration of 0.6%. Following GA exposure, the tissue is thoroughly rinsed to remove residual free aldehydes. The favorable balance of GA treated tissue between general structural integrity, intraoperative handling, and conformability has led to its continued widespread use (Jonas et al., 2002).

In a large pediatric cohort, GA-treated pericardial patches (0.625% for an incubation time of 7–10 min, selected at the discretion of the operating surgeon) were used for repairs of the aorta, pulmonary arteries, septal defects, and other sites. At a median follow-up of over 5 years, patch-related reoperation and intervention rates were low and no deaths were attributed to the patch itself (Gluck et al., 2022). While these results are comparable to other biomaterials used in similar clinical contexts, it is noteworthy that such critical treatment parameters as incubation time were left to surgical judgment rather than being standardized. This underscores a gap in evidence-based guidelines and highlights the importance of further mechanistic studies, like ours, that systematically investigate how GA treatment parameters influence structural and mechanical tissue behavior. These findings reinforce the notion that this is an active area of research with ongoing clinical relevance. Despite numerous studies on various treatment concentrations and tensile tests, comprehensive and high-quality data remain limited. The inherent challenges of analyzing such heterogeneous tissue complicate efforts to gain clear insights.

Rather than uncritically accepting established practices, it is important to evaluate their effects. The interpretation of the data must consider the limited sample size, particularly for mechanical testing, where only three fresh patient-derived specimens were available. Although such a small cohort restricts statistical power, the consistent treatment-related trends across donors suggest that the effects of GA concentration and incubation time are detectable despite biological variability.

While interaction effects between GA concentration and incubation time were identified at the ANOVA level, the absence of statistically significant *post hoc* differences suggests that these effects are subtle and influenced by biological variability. This indicates that GA treatment does not follow a simple linear dose–response relationship, but rather a coupled and non-additive interaction between concentration and exposure duration.

Variability between individuals plays a role because pericardial tissue properties are strongly influenced by patient-specific factors such as age, comorbidities, and intrinsic collagen/elastin composition. These biological differences inevitably contribute to variation in mechanical behavior. Nevertheless, in our dataset the treatment-related trends were consistent across the three donors, suggesting that the observed effects of GA concentration and incubation time are not purely driven by inter-individual variability.

Histological analyses were performed on both fresh surgical and cadaveric tissues, which may not allow perfect structural comparability to the fresh specimens used in mechanical testing. The combination of cadaveric and fresh-tissue histology with fresh-tissue mechanics provides complementary insights into how treatment conditions shape tissue structure and function. The chosen range of GA concentrations (0.1%–2.5%) and incubation times (5, 20 and 90 min) was selected to encompass both the clinically established standard (0.6% for 10 min, as used in the Ozaki procedure (Ozak et al., 2011)) and wider experimental conditions reported in the literature (Lee et al., 2017; Golomb et al., 1987; Poppenborg et al., 2022; Bezuidenhout et al., 2009), thereby enabling evaluation of whether deviations from current practice might further optimize tissue properties. Although a 10-min incubation condition was included in the initial cadaveric tissue screening, it was not retained for the final analysis in living tissue samples because the number of experimental conditions had to be prioritized due to limited availability of viable patient-derived tissue. We tested a total of 102 different combinations of treatment concentrations and incubation times on cadaveric (body donor) tissues. Untreated samples (0% GA) were used as the baseline reference and correspond to the 0 min condition across all analyses. It became clear that when transitioning to living tissue samples, a selection of these combinations needed to be prioritized due to the limited availability of viable tissue obtained from each patient. This focused subset was selected based on sample availability, consistency of imaging quality, and feasibility of direct comparison across treatment groups. Samples were harvested from the sternal side of the patients and carefully prepared to ensure uniform dimensions and good quality for testing. All processing steps followed standardized protocols to minimize variability.

Preliminary visual assessments of collagen fiber organization, based on histological staining and microscopy, already revealed observable differences between treatment conditions (Marcos-Garcés et al., 2022; Whittaker et al., 1994). Increasing GA concentration and incubation time were associated with reduced collagen waviness and increased fiber bundling, supporting the hypothesis of crosslinking-induced structural stiffening restricting fiber mobility. Attempts to statistically quantify these visual differences were limited by high inter-patient variability.

Notably, the tissue thickness exhibited inter-individual variability, and the presence of regional heterogeneity within individual samples further complicated data interpretation, as previously reported by Stieglmeier et al. (2021). The variability observed in control samples of mechanical parameters, orientation and waviness underscores the intrinsic heterogeneity of human pericardial tissue, which can lead to measurable differences even in the absence of treatment and highlights the importance of within-sample comparative approaches.

The inclusion of a within-person repeated-measures correlation analysis provided additional insight into the relationship between structural and mechanical parameters. The strongest positive correlation was observed between maximum stress and the high-strain elastic modulus (E_high_), indicating that stiffness in the high-strain region is a key determinant of load-bearing capacity. Similarly, threshold stress showed strong associations with the low-strain elastic modulus (E_low_), suggesting that early mechanical response is governed by low-strain stiffness properties. In contrast, structural parameters such as collagen waviness and fiber orientation showed negligible correlation (r_rm_ = 0.06), indicating that these features behave largely independently and may be affected differently by GA-induced crosslinking.

Uniaxial tensile testing is widely used in assessing the mechanical properties of heart valve leaflets and GA treated pericardium (Yamashita et al., 2012; Zioupos et al., 1994; Koechlin et al., 2021). This approach was therefore selected to enable direct comparison with previous studies and reproducibility of findings. While this method simplifies the physiological loading environment, the derived parameters (e.g., stiffness, strain) represent fundamental descriptors that also contribute to multiaxial tissue behavior. It is acknowledged that valve leaflets are not subjected to purely uniaxial loading *in vivo*. Therefore, the present findings should be interpreted as controlled, comparative insights, and future work should incorporate biaxial testing to better approximate physiological conditions.

The results showed that shorter incubation times appeared to induce early structural and mechanical alterations in the tissue, with the extent of these effects modulated by tissue thickness. These gradual trends were observed across multiple mechanical parameters. More consistent patterns were noticeable particularly after intermediate exposure durations.

Treatment with a 20-min incubation time, was associated with increased extensibility, higher peak load-bearing capacity ( $\left.\sigma_{\max}\right)$ , and improved resistance to deformation.

In contrast, prolonged incubation (90 min) was linked to more variable responses, potentially indicating early signs of tissue overprocessing. While not all trends reached statistical significance, the observed tendencies may indicate that increased stiffness at higher concentrations and intermediate incubation times corresponds to enhanced collagen crosslinking and reduced compliance.

Taken together, these findings indicate that the effects of GA treatment on pericardial tissue are not governed by a simple increase in crosslinking with higher concentration or longer incubation, but instead reflect a non-linear and coupled interaction between these parameters. Notably, structural descriptors such as collagen waviness and fiber orientation responded independently and did not directly correlate with mechanical outcomes, highlighting that commonly used structural metrics may not fully predict functional behavior in heterogeneous biological tissues.

The broader range of concentrations and incubation times tested in this study was chosen to place the clinically established standard of 0.6% GA for 10 min, as used in the Ozaki procedure, into a wider experimental context. Our results showed that this standard condition falls within the range where both structural preservation and mechanical stability were observed, suggesting it remains a reasonable balance between stiffness and compliance. However, the observed non-linear interaction between concentration and incubation time indicates that treatment effects cannot be predicted based on a single parameter alone. Trends at higher concentrations or prolonged incubation times indicate that further optimization may be possible, particularly with respect to fiber recruitment behavior. These findings therefore support current clinical practice while also motivating more systematic, parameter-driven optimization of treatment protocols.

Beyond the specific findings, the approach presented here may be useful for researchers facing similar methodological constraints associated with heterogeneous biological tissues. Balancing scientific rigor with practical feasibility is a persistent issue in tissue-based research. While this study is limited by sample variability and size, it nonetheless yields several meaningful insights. Our observations revealed potential treatment-related effects, fiber orientation was influenced by incubation time, and both maximum strain and transition strain demonstrated interaction effects between treatment concentration and incubation time.

Recent advances in mechanically adjustable biomaterials, including reinforced hydrogels highlight the growing interest in designing materials with improved structural mechanical matching for tissue engineering and cardiovascular applications, providing useful context for future biomaterial development and emphasize the importance of integrated structural and mechanical assessment (Jia et al., 2025).

The structured and reproducible methodology presented here provides a framework for integrating structural and mechanical analyses in heterogeneous tissues, particularly when sample availability is limited.

### 4.1 Limitations

The most significant limitation of this study is the small sample size, particularly for mechanical testing, where only tissues from three individual donors were included. As a result, the findings should be considered preliminary, and the limited statistical power likely contributed to the absence of significant *post hoc* differences despite significant ANOVA results.

Variability in the data is further influenced by the intrinsic heterogeneity of human pericardial tissue, both between donors and within different regions of the same specimen. This heterogeneity affects structural parameters such as fiber orientation and waviness and may obscure subtle treatment-related effects. While cadaveric tissue was included for histological assessment, mechanical testing was performed exclusively on fresh surgical specimens to ensure physiological relevance. This limits direct correlation between structural and mechanical datasets obtained from the same samples.

Practical constraints associated with human tissue collection also resulted in variability in sample processing and storage times prior to testing. Samples were prepared based on available tissue geometry rather than predefined collagen fiber orientation and although all specimens were maintained under controlled conditions, differences in storage duration may have contributed to variability in the measured mechanical properties. In addition, while GA concentration and incubation time were systematically varied, other potentially relevant parameters, such as temperature, pH stability, and post-treatment washing, were kept constant and not independently optimized.

Finally, mechanical characterization was limited to uniaxial tensile testing. Although this approach provides a well-controlled and widely used framework for assessing tissue mechanics, it does not fully capture the complex multiaxial loading conditions experienced *in vivo*. Consequently, direct translation of these results to physiological behavior is not possible without additional modeling or complementary testing approaches.

While repeated-measures correlation analysis provided insight into relationships between parameters, these associations remain observational and do not imply causation, and should therefore be interpreted as descriptive rather than mechanistic.

### 4.2 Conclusion

In summary, this study provides new and systemic insights into how varying GA concentrations and incubation times influence mechanical properties and fiber organization. Structural analysis showed a time-dependent reduction in fiber waviness, with increasing durations leading to more linear fiber configurations. This observation is consistent with reduced collagen crimp and increased structural stabilization under GA treatment. The observed fiber thickening and bundling further indicate treatment-induced microstructural reorganization. Uniaxial tensile testing revealed a significant interaction effect between incubation time and GA concentration on the maximum strain, indicating that the mechanical response is not governed by a non-linear and non-additive interaction between these parameters. A similar interaction effect between GA concentration and incubation time was observed for the transition strain, suggesting that the onset of collagen fiber recruitment during loading is modulated by both treatment parameters. These findings demonstrate that GA treatment affects not only overall stiffness but also the transition between mechanical regimes. Importantly, correlation analysis showed that structural descriptors such as collagen waviness and fiber orientation were not strongly associated with mechanical outcomes, indicating that commonly used structural metrics may not directly predict functional behavior in heterogeneous tissues. These findings support the hypothesis that GA treatment alters not only the ultimate mechanical strength but also the mechanical response profile and fiber recruitment behavior of pericardial tissue.

Overall, the results demonstrate that GA treatment effects cannot be predicted by concentration or incubation time alone, instead arise from their coupled interaction. This highlights the need for integrated structural-mechanical evaluation when optimizing treatment protocols for bioprosthetic applications.

Future studies will be required to determine whether these structural and mechanical findings translate into improved performance or biological safety.

## Data Availability

The original contributions presented in the study are included in the article/Supplementary Material, further inquiries can be directed to the corresponding author.
