# Supplementary material for: Evaluating the effects of glutaraldehyde concentration and incubation time on the structural integrity of the human pericardium
Source: Front Bioeng Biotechnol. 2026 Jun 17;14:1746518. doi: 10.3389/fbioe.2026.1746518 (PMC13320602; doi:10.3389/fbioe.2026.1746518)
Supplement: Supplementary file 1 [file DataSheet1.pdf]

Supplementary Figure 1

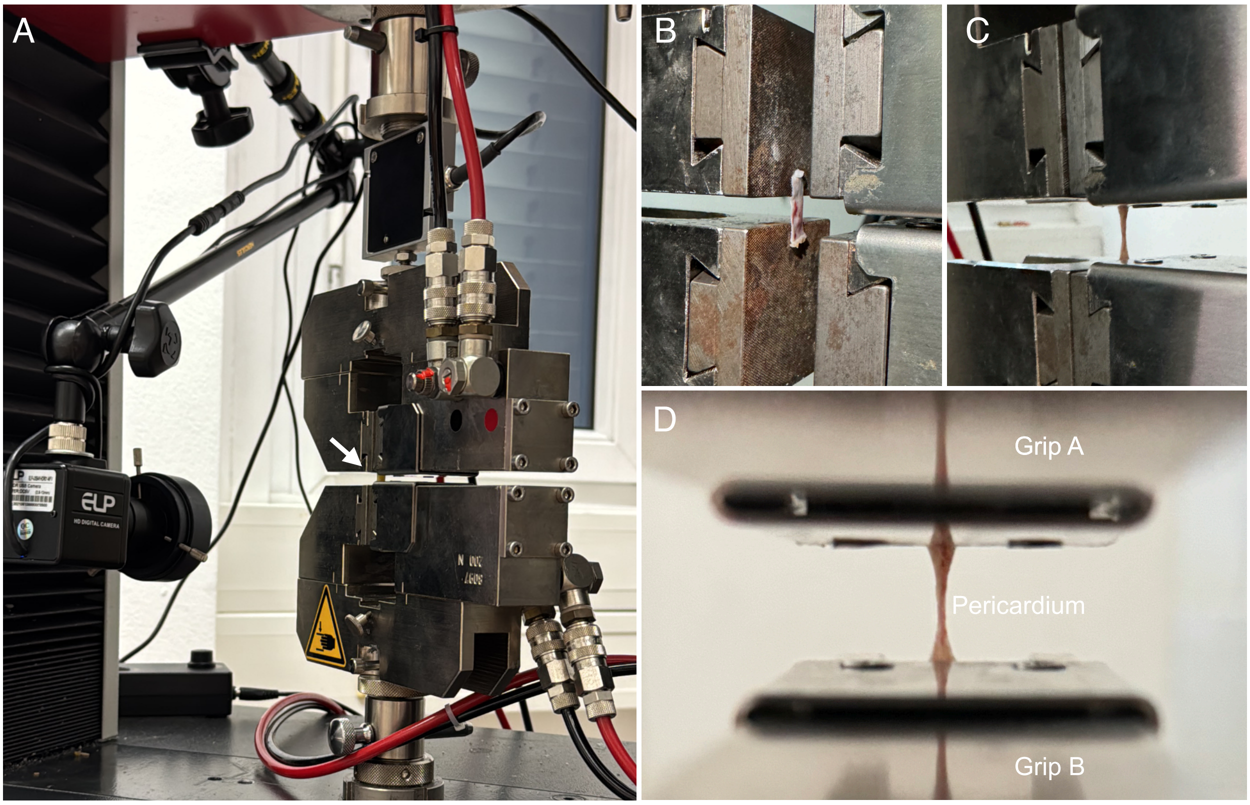

Supplementary Figure 1. Setup for Uniaxial Testing. (A) Experimental setup of the Zwick Roell Z005 uniaxial testing machine with two grips mounted on top of each other holding the specimen in between (marked with white arrow). (B) Rectangular sample positioned between the grips prior to loading, with no applied tension. (C) Side view of the sample clamped between closed grips during the test. (D) Frontal view of the sample (pericardium) under tension, illustrating uniform stretching during the experiment.

Supplementary Figure 2

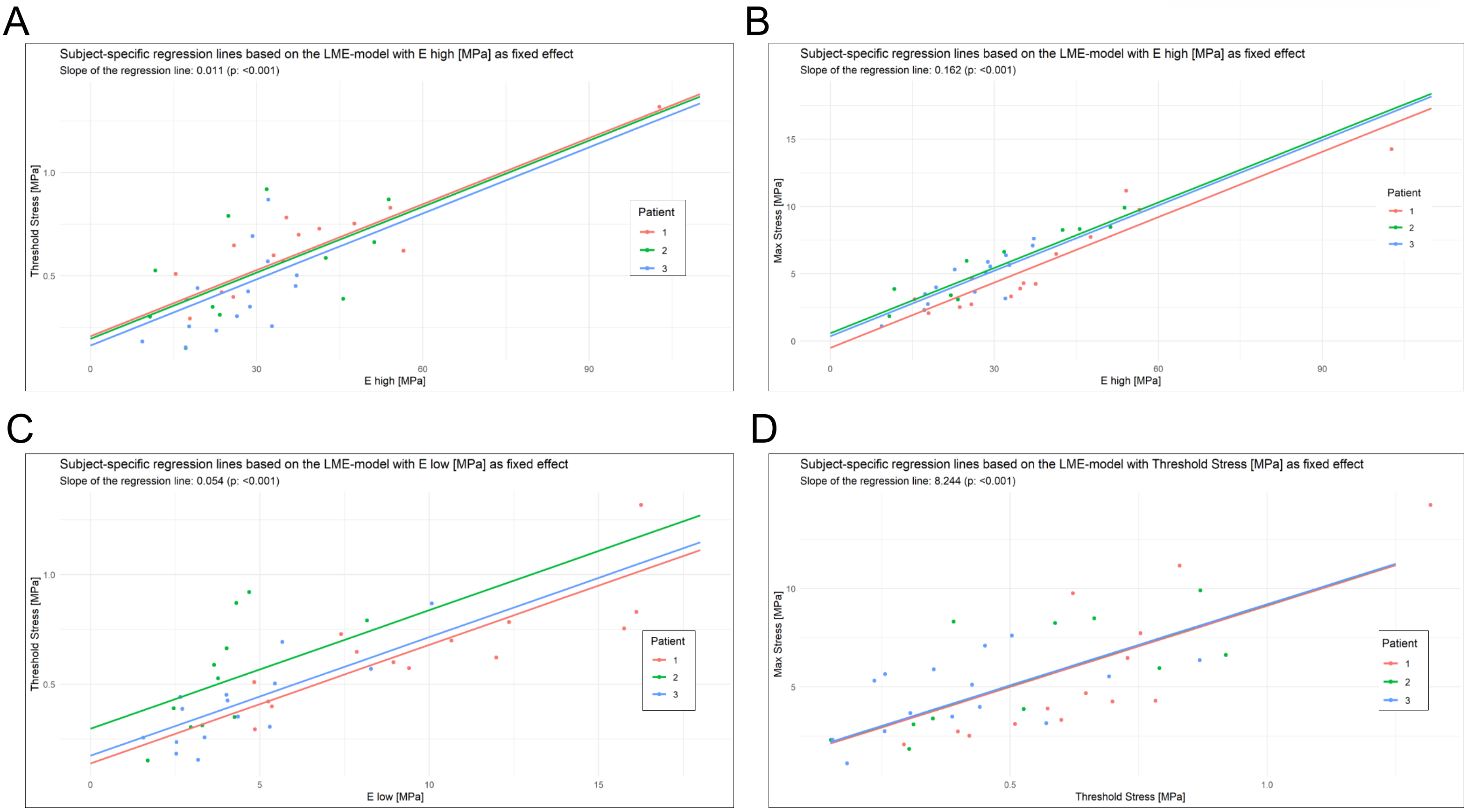

Supplementary Figure 2. Subject-specific regression analysis of mechanical parameter relationships.
Subject-specific regression lines derived from linear mixed-effects (LME) models illustrating relationships between selected mechanical parameters across all treatment conditions. Fixed effects were estimated at the population level, while subject-specific random effects account for inter-individual variability. The following parameter relationships are shown: A) threshold stress [MPa] versus high-strain elastic modulus
(E_high [MPa]), B) maximum stress [MPa] versus high-strain elastic modulus (E_high [MPa]), C) threshold stress [MPa] versus low-strain elastic modulus (E_low[MPa]), and D) maximum stress versus threshold stress. Each line represents an individual subject, highlighting within-subject trends, while the overall slope reflects the fixed-effect estimate from the LME model. These analyses complement the repeated-measures correlation results by illustrating the strongest within-subject relationships among mechanical parameters, which remain consistent despite inter-sample variability.

Supplementary Figure 3

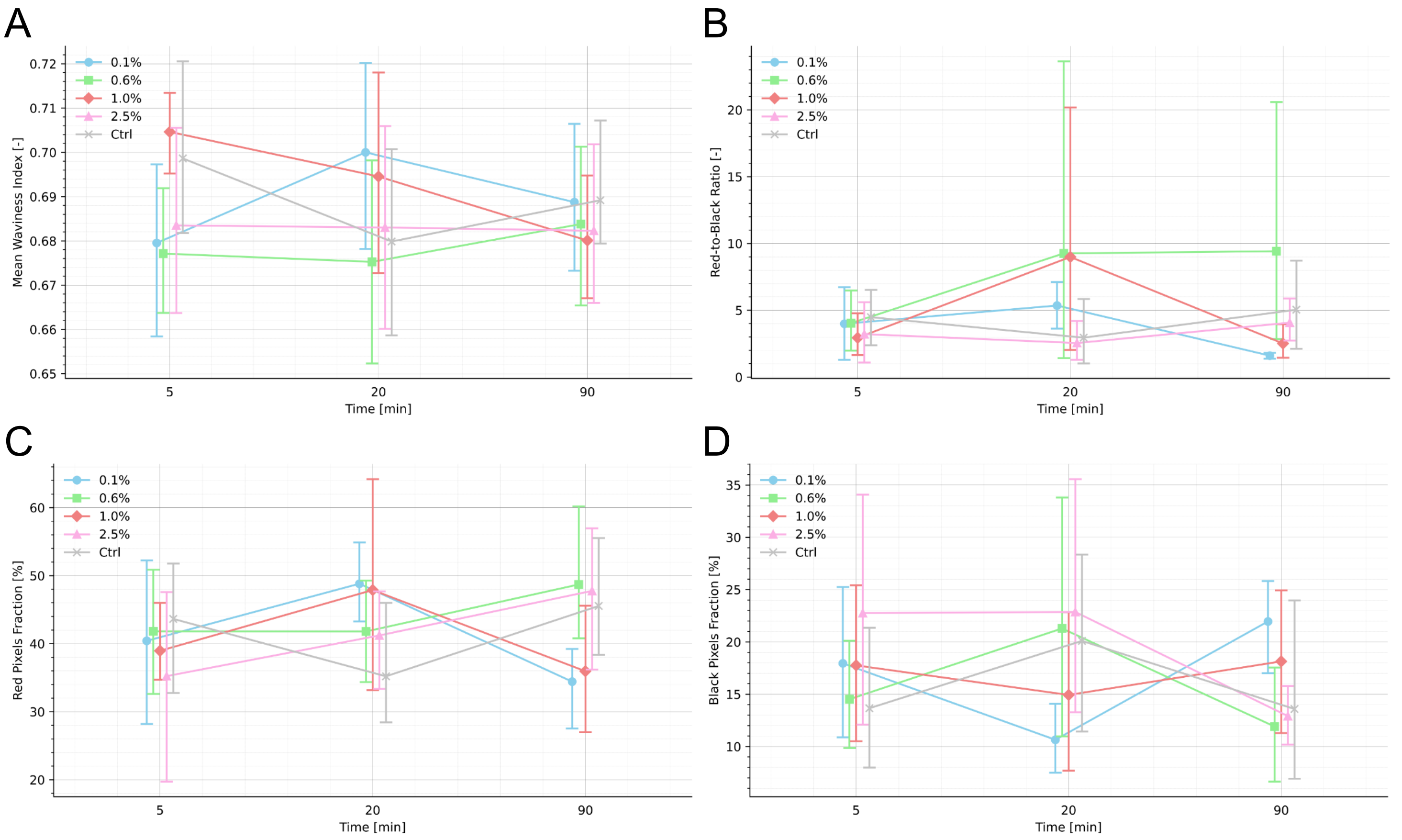

E

Parameter	Effect	df	F-Value	p-Value
**Waviness Index Mean**	GA [%]	4	0,83	0,51
**Waviness Index Mean**	Incubation time [min]	2	0,12	0,88
**Waviness Index Mean**	Interaction (Time × GA)	8	0,75	0,65
**Waviness Index Mean**	Residual	69	-	-
**Red Pixels Fraction**	GA [%]	4	0,16	0,96
**Red Pixels Fraction**	Incubation time [min]	2	0,46	0,63
**Red Pixels Fraction**	Interaction (Time × GA)	8	1,26	0,28
**Red Pixels Fraction**	Residual	69	-	-
**Black Pixels Fraction**	GA [%]	4	0,32	0,86
**Black Pixels Fraction**	Incubation time [min]	2	0,30	0,74
**Black Pixels Fraction**	Interaction (Time × GA)	8	1,39	0,22
**Black Pixels Fraction**	Residual	69	-	-
**Red-to-Black Ratio**	GA [%]	4	0,94	0,44
**Red-to-Black Ratio**	Incubation time [min]	2	0,69	0,51
**Red-to-Black Ratio**	Interaction (Time × GA)	8	0,59	0,78
**Red-to-Black Ratio**	Residual	69	-	-

Supplementary Figure 3. Interaction Plots and Statistical Analysis of Image-Derived Pericardium Metrics. (A–D) Interaction plots showing the combined effects of glutaraldehyde (GA) treatment concentration (Control (Ctrl)…grey and marked with x; 0.1%…blue and marked with ●; 0.6%…green and marked with ■; 1.0%...red marked with ◆ and 2.5%...pink marked with ▲) and incubation time (5-, 20- and 90 minutes) on image-based measurements: (A) Mean waviness index, (B) Red-to-black pixel intensity ratio, (C) Fraction of red pixels, and (D) Fraction of black pixels. Each plot illustrates how the measured parameter varies across treatment conditions, with separate lines representing different incubation times. Each condition includes measurements from n = 6 samples. (E) Summary table of the statistical analysis (two-way ANOVA), reporting the main effects (GA concentration [%], incubation time [min]), their interaction (time × concentration), and associated degrees of freedom (df), F-values, and p-values for each parameter. Asterisks (*) indicate statistically significant effects ($p < 0.05$).

Supplementary Figure 4

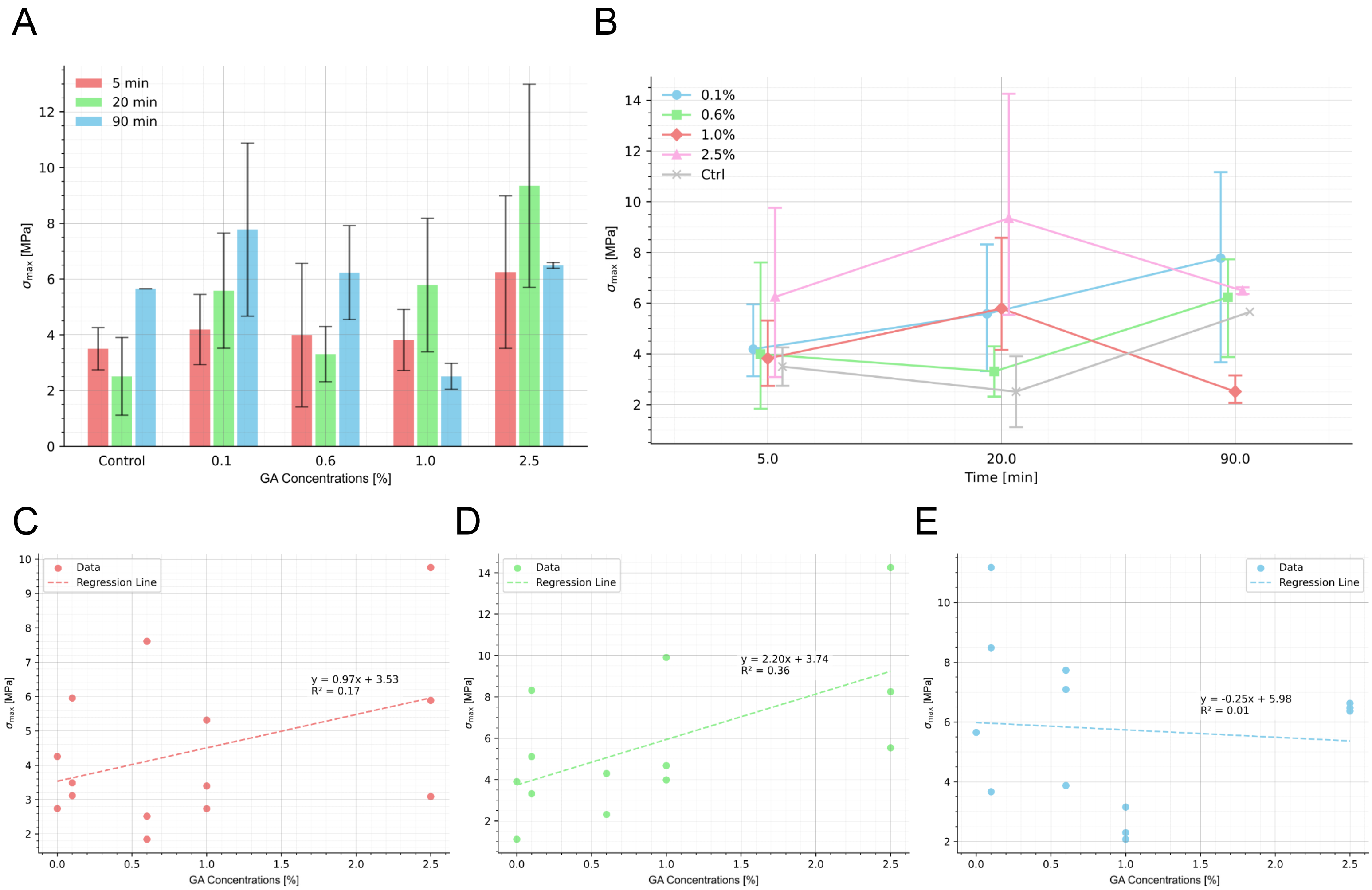

F

Effect	df	F-Value	p-Value
Incubation time [min]	2.0	1.31	0.29
GA [%]	3.0	2.66	0.07
Interaction (Time × GA)	6.0	1.22	0.33
Residual	24.0	–	–

G

Time Point	Slope	Intercept	R^2 Score
5 minutes	0.91	3.53	0.166
20 minutes	2.23	3.68	0.374
90 minutes	-0.25	5.98	0.008

Supplementary Figure 4. Tensile Testing Analysis of Maximum Stress Across Treatment Conditions.
(A) Bar plots showing the mean maximum stress measured via tensile testing for each Glutaraldehyde (GA) concentration, pooled across all incubation times (5 minutes…red; 20 minutes…green; 90 minutes…blue). Error bars represent variability (standard deviation) across n = 3 samples per condition. **(B)** Interaction plot illustrating the combined effects of GA concentration (Control (Ctrl)…grey and marked with x; 0.1%…blue and marked with ●; 0.6%…green and marked with ■; 1.0%...red marked with ◆ and 2.5%...pink marked with ▲) and incubation time on maximum stress. **(C–E)** Linear regression plots showing the relationship between GA concentration and maximum stress for each incubation time: **(C)** 5 minutes, **(D)** 20 minutes, **(E)** 90 minutes. Each plot includes a regression line indicating the trend in maximum stress with increasing GA concentration. **(F)** Two-way ANOVA summary table for the interaction plot in panel **(B)**, showing the effects of GA concentration, incubation time, and their interaction (time × concentration) on maximum stress. **(G)** Regression analysis results from panels **(C–E)**, including slope, intercept, and coefficient of determination (R^2) for each incubation time. Slope is expressed as % strain per % concentration. R^2 is unitless.

Supplementary Figure 5

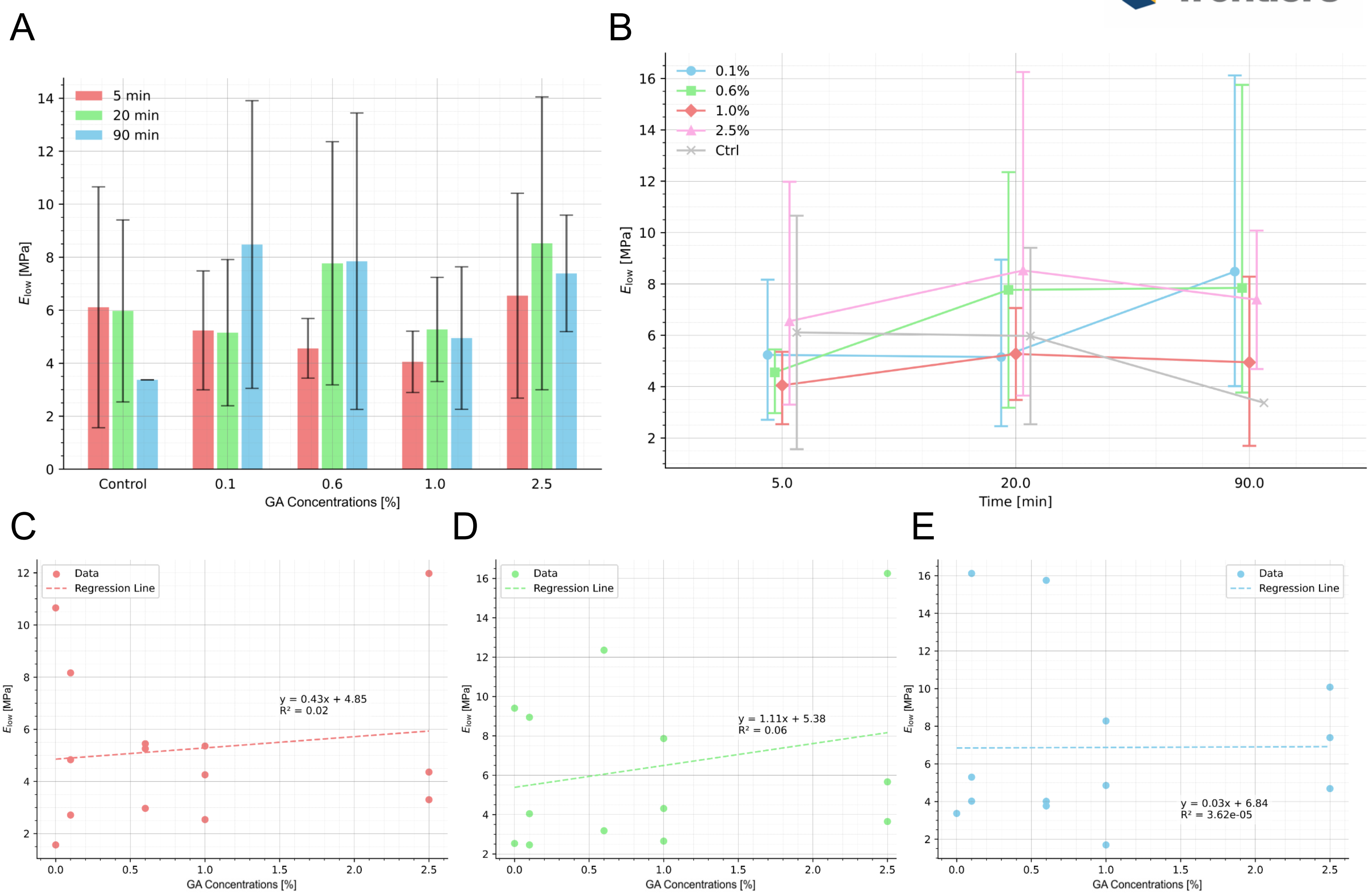

F

Effect	df	F-Value	p-Value
Incubation time [min]	2.0	0.72	0.50
GA [%]	3.0	0.64	0.60
Interaction (Time × GA)	6.0	0.20	0.97
Residual	24.0	–	–

G

Time Point	Slope	Intercept	R^2Score
5 minutes	0.43	4.85	0.018
20 minutes	1.23	5.29	0.078
90 minutes	0.03	6.84	3.6×10^{-5}

Supplementary Figure 5. Tensile Testing Analysis of Elastic Modulus (Low-Strain Region) Across Treatment Conditions. **(A)** Bar plots showing the mean elastic modulus (low-strain region) for each Glutaraldehyde (GA) concentration, pooled across all incubation times (5 minutes…red; 20 minutes…green; 90 minutes…blue). Error bars represent variability (standard deviation) across *n* = 3 samples per condition. **(B)** Interaction plot illustrating the combined effects of GA concentration (Control (Ctrl)…grey and marked with x; 0.1%…blue and marked with ●; 0.6%…green and marked with ■; 1.0%...red marked with ◆ and 2.5%...pink marked with ▲) and incubation time on elastic modulus$_{low}$. **(C–E)** Linear regression plots showing the relationship between GA concentration and elastic modulus$_{low}$ for each incubation time: **(C)** 5 minutes, **D)** 20 minutes, **E)** 90 minutes. Each plot includes a regression line indicating the trend in the elastic modulus$_{low}$ with increasing GA concentration. **(F)** Two-way ANOVA summary table for the interaction plot in panel (B), showing the effects of GA concentration, incubation time, and their interaction (time × concentration) on the elastic modulus$_{low}$. **(G)** Regression analysis results from panels (C–E), including slope, intercept, and coefficient of determination (R^2) for each incubation time. Slope is expressed as % strain per % concentration. R^2 is unitless. Values were obtained from tensile testing.

Supplementary Figure 6

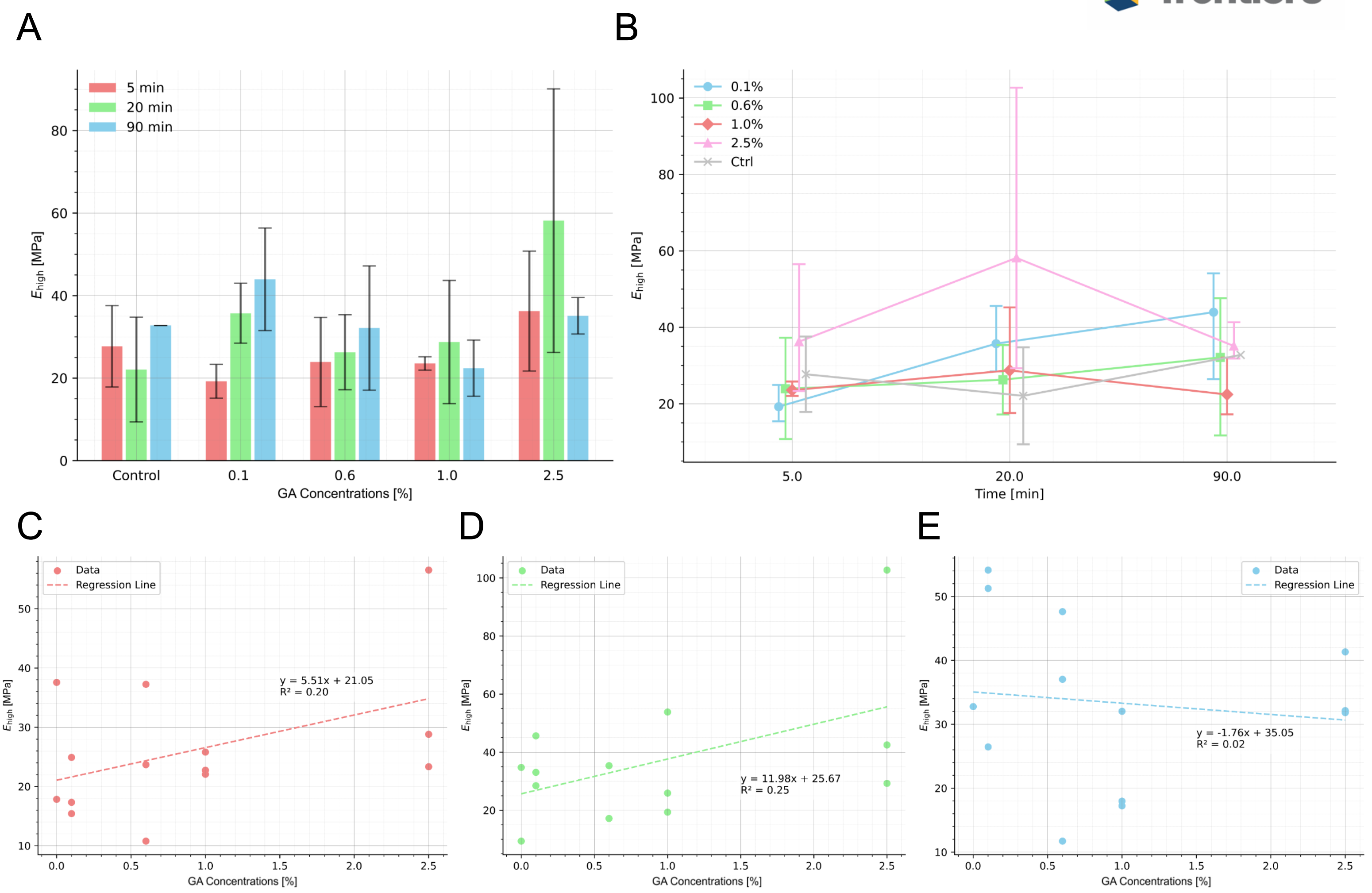

F

Effect	df	F-Value	p-Value
Incubation time [min]	2.0	1.58	0.23
GA [%]	3.0	2.12	0.12
Interaction (Time × GA)	6.0	0.76	0.60
Residual	24.0	–	–

G

Time Point	Slope	Intercept	R^2 Score
5 minutes	5.51	21.05	0.202
20 minutes	12.30	24.16	0.254
90 minutes	-1.76	35.05	0.016

Supplementary Figure 6. Tensile Testing Analysis of Elastic Modulus (High-Strain Region) Across Treatment Conditions. **(A)** Bar plots showing the mean elastic modulus (low-strain region) for each Glutaraldehyde (GA) concentration, pooled across all incubation times (5 minutes…red; 20 minutes…green; 90 minutes…blue). Error bars represent variability (standard deviation) across *n* = 3 samples per condition. **(B)** Interaction plot illustrating the combined effects of GA concentration (Control (Ctrl)…grey and marked with x; 0.1%…blue and marked with ●; 0.6%…green and marked with ■; 1.0%...red marked with ◆ and 2.5%...pink marked with ▲) and incubation time on elastic modulus$_{high}$. **(C–E)** Linear regression plots showing the relationship between GA concentration and elastic modulus$_{high}$ for each incubation time: **(C)** 5 minutes, **D)** 20 minutes, **E)** 90 minutes. Each plot includes a regression line indicating the trend in the elastic modulus$_{high}$ with increasing GA concentration. **(F)** Two-way ANOVA summary table for the interaction plot in panel (B), showing the effects of GA concentration, incubation time, and their interaction (time × concentration) on the elastic modulus$_{high}$. **(G)** Regression analysis results from panels (C–E), including slope, intercept, and coefficient of determination (R^2) for each incubation time. Slope is expressed as % strain per % concentration. R^2 is unitless. Values were obtained from tensile testing.

Supplementary Figure 7

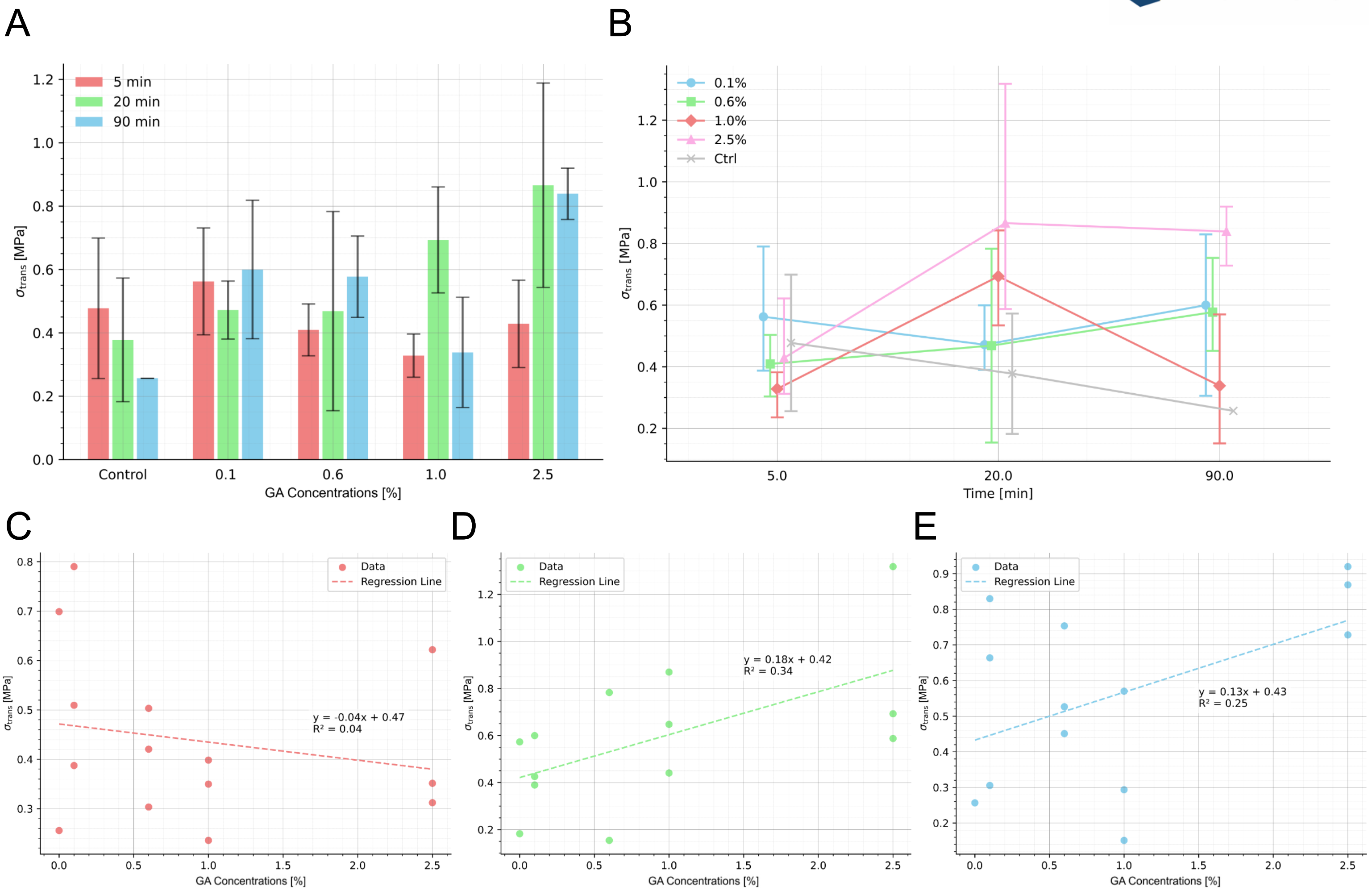

F

Effect	df	F-Value	P-Value
Incubation time [min]	2.0	3.12	0.06
GA [%]	3.0	2.28	0.10
Interaction (Time × GA)	6.0	1.59	0.19
Residual	24.0	–	–

G

Time Point	Slope	Intercept	R^2Score
5 minutes	-0.04	0.47	0.043
20 minutes	0.19	0.44	0.353
90 minutes	0.13	0.43	0.246

Supplementary Figure 7. Effect of Treatment Time and Concentration on Transition Stress in Tensile Testing.
(A) Barplot showing the transition stress (σ_{trans}) across different Glutaraldehyde (GA) concentrations. Each bar represents the mean from all patients treated for 5 minutes (red), 20 minutes (green), or 90 minutes (blue) at the respective GA concentrations. Error bars indicate variability (standard deviation) across patients (n=3). **(B)** Interaction plot showing the effects of treatment time and concentration (Control (Ctrl)…grey and marked with x; 0.1%…blue and marked with ●; 0.6%…green and marked with ■; 1.0%...red marked with ◆ and 2.5%...pink marked with ▲) on transition strain. Each data point represents the mean of three biological replicates (patient samples), the error bars show the standard deviation. **(C–E)** Linear regression plots showing the relationship between GA concentration and transition stress (σ_{trans}) for each incubation time: **(A)** 5 minutes (red), **(B)** 20 minutes (green),
(C) 90 minutes (blue). In each plot, the x-axis represents increasing GA concentrations, and the y-axis shows the corresponding transient strain. Trend lines illustrate the linear relationship for each time point. **(D)** Summary table of regression parameters, including slope, intercept, and coefficient of determination (R^2) for each incubation time. Slope is expressed as % strain per % concentration. R^2 is unitless.

Supplementary Table 1

Patient	Concentration (%)	Height (mm)	Width (mm)	Thickness (mm)	Area [mm^2]
1	0,10	14,00	6,00	0,57	3,41
1	0,10	13,00	5,00	0,75	3,76
1	0,10	14,00	4,00	0,44	1,77
1	0,60	15,00	6,00	0,44	2,62
1	0,60	15,00	5,00	0,36	1,81
1	0,60	14,00	5,00	0,57	2,84
1	1,00	15,00	6,00	0,64	3,84
1	1,00	15,00	7,00	0,86	5,99
1	1,00	14,00	4,00	0,76	3,03
1	2,50	12,00	5,00	0,52	2,59
1	2,50	12,00	5,00	0,35	1,73
1	2,50	12,00	5,00	0,29	1,46
1	Ctrl	15,00	5,00	0,51	2,53
1	Ctrl	10,00	6,00	0,48	2,85
1	Ctrl	-	-	-	-
2	0,10	12	4	0,95	3,81
2	0,10	9	3	0,72	2,17
2	0,10	12	3	0,73	2,20
2	0,60	10	5	0,86	4,28
2	0,60	10	4	0,60	2,40
2	0,60	10	3	1,13	3,39
2	1,00	9	5	0,92	4,59
2	1,00	12	4	0,75	3,01
2	1,00	10	5	0,86	4,31
2	2,50	12	5	1,00	5,01
2	2,50	12	3	1,04	3,12
2	2,50	12	4	0,85	3,40
2	Ctrl	12	4	0,64	2,56
2	Ctrl	12	4	0,76	3,02
2	Ctrl	12	4	0,90	3,60
3	0,10	11	6	0,74	4,42
3	0,10	12	5	0,67	3,35
3	0,10	11	6	0,63	3,76
3	0,60	11	7	0,71	4,95
3	0,60	11	4	1,31	5,23
3	0,60	12	5	0,72	3,59
3	1,00	13	5	0,74	3,70
3	1,00	13	7	1,04	7,28
3	1,00	11	7	0,52	3,65
3	2,50	13	5,5	1,27	6,98
3	2,50	11	5	0,88	4,41
3	2,50	10	5	0,49	2,47
3	Ctrl	9	6	1,04	6,23
3	Ctrl	12,5	6	0,88	5,25
3	Ctrl	13	6	0,85	5,11

Supplementary Table 1. Pericardial Tissue Sample Dimensions for Tensile Testing.
The table lists individual pericardial tissue samples used for tensile testing, identified by patient number (1–3), GA concentration (including control, abbreviated as "Ctrl" for 1X PBS), and corresponding sample dimensions: height (mm), width (mm), thickness (mm), and calculated cross-sectional area (mm^2). These measurements were recorded prior to mechanical testing.

Supplementary Table 2

		Max strain [%]	Max Stress [Mpa]	E low [Mpa]	E high [Mpa]	Trans Strain [%]	Trans Stress [Mpa]
Ctrl	5 min	24.25 (5.25)	3.50 (0.76)	6.11 (4.55)	27.71 (9.87)	11.55 (2.95)	0.48 (0.22)
	20 min	19.6 (0.79)	2.51 (1.40)	5.97 (3.44)	22.07 (12.69)	8.6 (0.5)	0.38 (0.19)
	90 min	27.5 (/)	5.65 (/)	3.37 (/)	32.77 (/)	8.7 (/)	0.26 (/)
0.10%	5 min	32.1 (1.57)	4.19 (1.26)	5.24 (2.24)	19.22 (4.10)	12.30 (1.34)	0.56 (0.17)
	20 min	26.43 (6.47)	5.58 (2.07)	5.15 (2.76)	35.72 (7.25)	10.6 (1.49)	0.47 (0.09)
	90 min	26.37 (3.31)	7.77 (3.10)	8.48 (5.43)	43.94 (12.42)	9.30 (2.90)	0.60 (0.22)
0.60%	5 min	25.40 (4.56)	3.99 (2.57)	4.56 (1.13)	23.91 (10.81)	10.37 (0.54)	0.41 (0.08)
	20 min	20.35 (1.05)	3.31 (0.99)	7.77 (4.59)	26.28 (9.08)	7.6 (0.8)	0.47 (0.31)
	90 min	30.40 (6.43)	6.23 (1.69)	7.85 (5.59)	32.12 (15.06)	10.23 (2.60)	0.58 (0.13)
1.00%	5 min	26.60 (6.57)	3.82 (1.09)	4.05 (1.16)	23.54 (1.63)	9.70 (0.45)	0.33 (0.07)
	20 min	32.73 (4.55)	5.79 (2.39)	5.27 (1.97)	28.73 (14.93)	13.28 (2.21)	0.69 (0.17)
	90 min	20.23 (2.59)	2.51 (0.46)	4.95 (2.69)	22.41 (6.81)	8.67 (0.45)	0.34 (0.17)
2.50%	5 min	25.93 (2.98)	6.25 (2.73)	6.54 (3.86)	36.23 (14.53)	8.9 (1.36)	0.43 (0.14)
	20 min	29.17 (4.32)	9.35 (3.64)	8.52 (5.53)	58.15 (31.95)	12.20 (2.05)	0.87 (0.32)
	90 min	30.67 (5.78)	6.48 (0.11)	7.39 (2.20)	35.09 (4.41)	13.27 (3.78)	0.84 (0.08)

Supplementary Table 2. Mechanical Properties Derived from Tensile Testing.
Calculated parameters from tensile testing for each sample. Reported values include: Maximum strain [%], Maximum stress [Mpa], Elastic modulus at low strain (E low, MPa), Elastic modulus at high strain (E high, MPa), Transition strain [%] and Transition stress [Mpa]. Values are presented as mean ± standard deviation (n=3 per condition). All parameters were derived from the corresponding stress–strain curves obtained during uniaxial tensile testing.
